# Regulation Network of Colorectal-Cancer-Specific Enhancers in the Progression of Colorectal Cancer

**DOI:** 10.3390/ijms22158337

**Published:** 2021-08-03

**Authors:** Bohan Chen, Yiping Ma, Jinfang Bi, Wenbin Wang, Anshun He, Guangsong Su, Zhongfang Zhao, Jiandang Shi, Lei Zhang

**Affiliations:** State Key Laboratory of Medicinal Chemical Biology and College of Life Sciences, Nankai University, 94 Weijin Road, Tianjin 300071, China; 1120200511@mail.nankai.edu.cn (B.C.); 1120200510@mail.nankai.edu.cn (Y.M.); 1120180423@mail.nankai.edu.cn (J.B.); 2120181086@mail.nankai.edu.cn (W.W.); 1120160377@mail.nankai.edu.cn (A.H.); 819085@nankai.edu.cn (G.S.); zhfzhao@nankai.edu.cn (Z.Z.); shijd@nankai.edu.cn (J.S.)

**Keywords:** colorectal-cancer-specific enhancer, long-range interaction, Hi-C, HiChIP, TAD

## Abstract

Enhancers regulate multiple genes via higher-order chromatin structures, and they further affect cancer progression. Epigenetic changes in cancer cells activate several cancer-specific enhancers that are silenced in normal cells. These cancer-specific enhancers are potential therapeutic targets of cancer. However, the functions and regulation networks of colorectal-cancer-specific enhancers are still unknown. In this study, we profile colorectal-cancer-specific enhancers and reveal their regulation network through the analysis of HiChIP data that were derived from a colorectal cancer cell line and Hi-C and RNA-seq data that were derived from tissue samples by in silico analysis and in vitro experiments. Enhancer–promoter loops in colorectal cancer cells containing colorectal-cancer-specific enhancers are involved in more than 50% of the topological associated domains (TADs) changed in colorectal cancer cells compared to normal colon cells. In addition, colorectal-cancer-specific enhancers interact with 152 genes that are significantly and highly expressed in colorectal cancer cells. These colorectal-cancer-specific enhancer target genes include *ITGB4*, *RECQL4*, *MSLN*, and *GDF15*. We propose that the regulation network of colorectal-cancer-specific enhancers plays an important role in the progression of colorectal cancer.

## 1. Introduction

Colorectal cancer is the world’s fourth most deadly cancer [1]. Previous studies on colorectal cancer have indicated that the sequential accumulation of genetic mutations and chromosomal instability are the most important reasons for the occurrence and development of colorectal cancer [2]. A typical genomic event in the initiation of colorectal cancer is *APC* mutation, followed by *RAS* activation or the function loss of *TP53* [1]. Additionally, the *MAPK*, *WNT*, *PI3K*, and TGF-β signaling pathways have been reported to play important roles in the initiation and progression of colorectal cancer [2,3]. Chromosomal changes including deletion, amplification, and translocation induce alterations in gene expression and function that may further initiate colorectal cancer. Studies on higher-order chromatin structures have revealed that long-range gene regulation networks are important in modulating biological processes in cells [4,5,6]. Further studies on gene regulation networks may help to unveil the occurrence and progression mechanisms of colorectal cancer.

**Figure 1:**
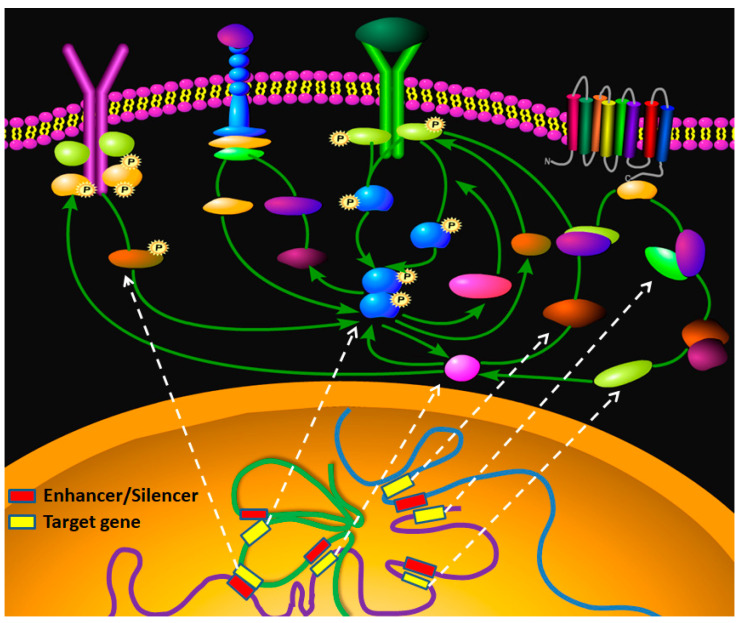
Schematic diagram of regulation networks of enhancers/silencers through higher-order chromatin structure in cancer cells. Enhancers/silencers regulate target genes through higher-order chromatin structure and affect the expressions of proteins in signaling pathways and further function in cancer progression.

Gene regulatory elements such as enhancers or silencers can regulate multiple target genes through higher-order chromatin structures [7,8] and functions in gene regulation networks in cancer cells (Figure 1). Mutations in gene regulatory elements and changes in higher-order chromatin structures are related to gene transcription and cancer progression [8,9,10]. A previous study revealed reorganizations of chromatin structures in colorectal cancers in comparison to normal colons, and the topological changes of chromatins were found to restrain the malignant progression of colorectal cancers [11]. In addition, changes of higher-order chromatin structures in colorectal cancer cells are accompanied by epigenetic changes such as alterations in DNA methylation [11,12]. These epigenetic changes are associated with changes in the binding of transcription factors and the activation of some gene regulatory elements such as enhancers. Some enhancers are activated in cancer cells but not in normal cells [13]. These “cancer-specific enhancers” may regulate several target genes through higher-order chromatin structures and play roles in cancer progression. These cancer-specific enhancers are potential therapeutic targets of cancer. However, how colorectal-cancer-specific enhancers function in cancer initiation and progression is still not very clear.

In this paper, we show the profiles of colorectal-cancer-specific enhancers and their gene regulation networks through the mining of databases and the combination analysis of Hi-ChIP data that were derived from a colorectal cancer cell line and Hi-C and RNA-seq data that were derived from tissue samples. The results of this study suggest that colorectal-cancer-specific enhancers can regulate multiple target genes including cancer-driver genes, as well as that genes regulated by these colorectal-cancer-specific enhancers are associated with the occurrence and development of colorectal cancer.

## 2. Results

### 2.1. H3K27ac Profiles Define Colorectal-Cancer-Specific Enhancers

**Figure 2:**
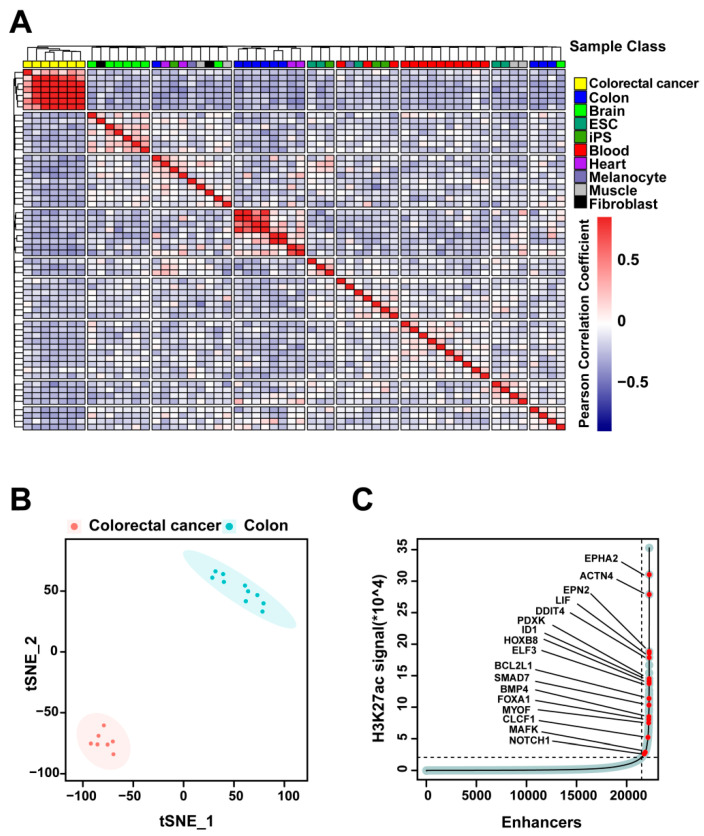
H3K27ac profiles define colorectal-cancer-specific enhancers. (**A**) The unsupervised hierarchical clustering of the 11,463 enhancer loci detected in colorectal-cancer samples (*n* = 7) compared to that of normal tissue samples (*n* = 10). (**B**) *t*-distributed stochastic neighbor embedding (*t*-SNE) analysis of normal colon-tissue-specific enhancers (Colon) and colorectal-cancer-specific enhancers. *t*-SNE_1 *p* = 2.03 × 10^−^^15^, *t*-SNE_2 *p* = 8.54 × 10^−^^12^. (**C**) Inflection plot showing the identified super enhancers among the cancer-specific enhancers.

H3K27ac is a well-characterized histone modification marker of active enhancers [14]. To identify colorectal-cancer-specific enhancers, we analyzed published H3K27ac CHIP-seq datasets from seven colorectal cancer datasets and ten normal tissue datasets from Roadmap Epigenomics [15], ENCODE [16], and a previously published study [17]. Unsupervised hierarchical clustering was performed on H3K27ac enrichment loci, identifying 10 clusters from the 59 samples (7 colorectal cancer samples, 10 normal colon samples, 8 brain samples, 5 ESC samples, 4 IPS samples, 13 blood samples, 4 heart samples, 2 melanocyte samples, 4 muscle samples, and 2 fibroblast samples from Roadmap Epigenomics [15], ENCODE [16], and a previously published study [17]) (Figure 2A) and showing that the distribution pattern of enhancers is highly tissue-specific, which is consistent with previous studies [13]. We identified 21,132 normal colon-tissue-specific enhancers and 11,463 colorectal-cancer-specific enhancers, and the *t*-distributed stochastic neighbor embedding (*t*-SNE) analysis indicated that colorectal cancer enhancer profiles are distinct from normal colon tissue enhancers (Figure 2B). In addition, we identified 763 super enhancers among the colorectal-cancer-specific enhancers (Figure 2C). Some of these super enhancers are associated with oncogenes, such as *EPHA2*, *LIF*, *ID1*, *SMAD7*, *BMP4*, *FOXA1*, and *NOTCH1*. Super enhancers are characterized as large clusters of enhancers in close proximity, and they are required for the maintenance of cell identity [14,18].

### 2.2. HiChIP Identifies Chromatin Interactions Containing Colorectal-Cancer-Specific Enhancers

**Figure 3:**
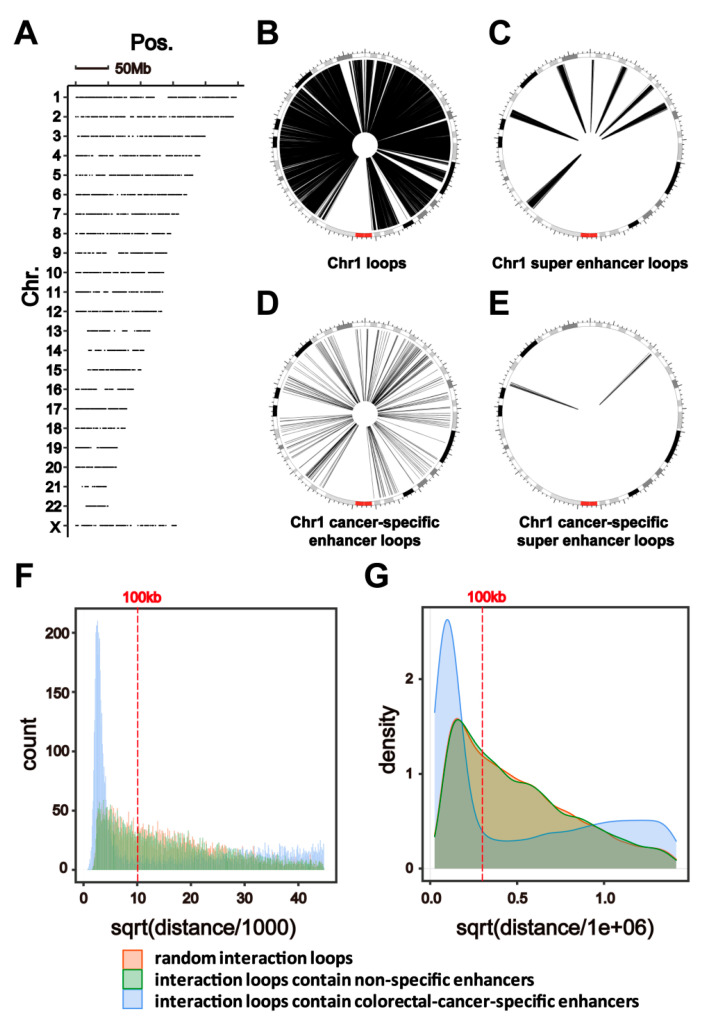
HiChIP identified chromatin interactions containing colorectal-cancer-specific enhancers. (**A**) Distributions of colorectal-cancer-specific enhancers of the human genome (hg19). (**B**,**C**) Circos plot showing interactions in Chr1, as indicated by curves extending from enhancers and super enhancers in colorectal cancer cells. Each curve in the Circos plot indicates one interaction loop that contains enhancers or super enhancers. (**D**,**E**) Circos plot showing interactions in Chr1, as indicated by curves extending from colorectal-cancer-specific enhancers and super enhancers in colorectal cancer cells. Each curve in the Circos plot indicates one interaction loop that contains colorectal-cancer-specific enhancers or super enhancers. (**F**,**G**) An analysis of the length distribution of random interaction loops from HiChIP data (red), the interaction loops that contain colorectal-cancer-non-specific enhancers (green) and colorectal-cancer-specific enhancers (blue, *p* < 2.2 × 10^−^^16^ compared with the other two groups).

The positions of colorectal-cancer-specific enhancers are shown in Figure 3A; these colorectal-cancer-specific enhancers are evenly distributed on each chromosome (Figure 3A). To identify the regulation network of colorectal-cancer-specific enhancers, HiChIP for H3K27ac was performed in the HCT116 colorectal cancer cell line. The HiChIP data indicated interaction loops between enhancers/super enhancers and other chromatin fragments (in Figure 3B,C, interaction loops in chromosome 1 are shown as examples). Additionally, colorectal-cancer-specific enhancers/super enhancers interact with several chromatin fragments, including some long-range chromatin regions (in Figure 3D,E, the interaction loops in chromosome 1 are shown as examples). Most of the interaction loops that contain colorectal-cancer-specific enhancers are less than 100 kb in length and include more short-range interactions than the random interaction loops from the HiChIP data (Figure 3F,G). In addition, the interaction loops that were measured to be more than 100 kb in length—which have lower proportions (Figure 3F,G), but longer interaction loops (>100 kb)—that contain colorectal-cancer-specific enhancers did not have a significantly lower proportion (Figure 3F,G), which suggests that more long-range interactions are associated with colorectal-cancer-specific enhancers. 

### 2.3. Change in TAD Boundaries in Colorectal Cancer Cells Compared to Normal Colon Cells

**Figure 4:**
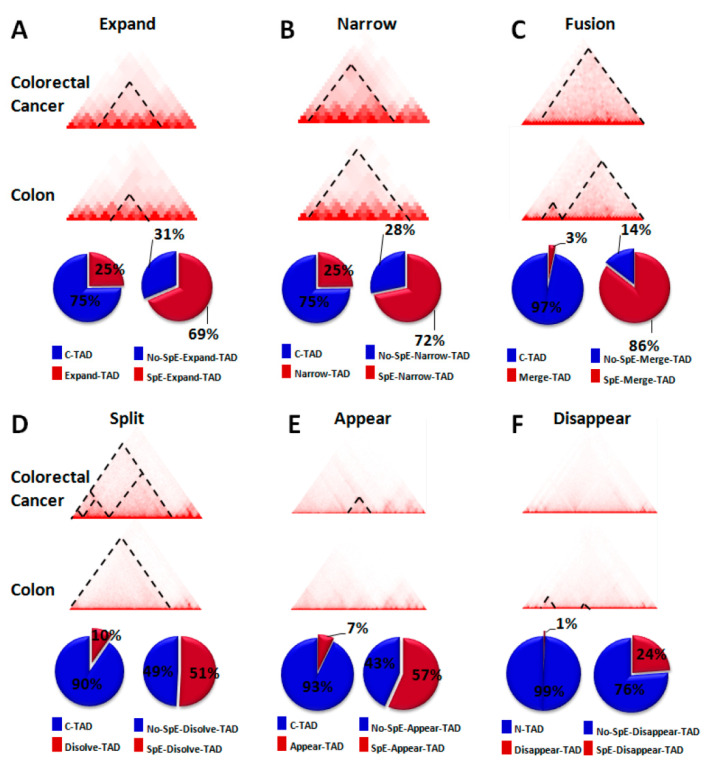
Changes of TAD boundaries in colorectal cancer cells compared to normal colon cells. (**A**) Expanded TADs in colorectal cancer cells compared to normal colon cells. (**B**) Narrowed TADs in colorectal cancer cells compared to normal colon cells. (**C**) Fusion TADs in colorectal cancer cells, where two or more TADs in normal colon cells are fused to one TAD in colorectal cancer cells. (**D**) Split TADs in colorectal cancer cells, where new insulation boundaries are formed in one TAD in colorectal cancer cells. (**E**) Appeared TADs in colorectal cancer cells that do not exist in normal colon cells. (**F**) Disappeared TADs, which are TADs in normal colon cells that disappear in colorectal cancer cells. The pie charts (**A**–**F**) show the percentages of specific categories of boundary-changed TADs (category-TAD) in the total changed TADs (C-TAD) in colorectal cancer cells (the first pie chart in each panel) and the percentages of specific categories of boundary-changed TADs containing colorectal-specific enhancers (SpE-category-TAD) in the total changed TADs of this category (the second pie chart in each panel).

A comparison to normal colon cells and an analysis of Hi-C data in colorectal cancer cells and normal colon cells [11] showed that about 65% of the boundaries of TADs changed in colorectal cancer cells (Figure 4A–F). The boundary-changed TADs were divided into six categories—expand (Figure 4A), narrow (Figure 4B), fusion (Figure 4C), split (Figure 4D), appear (Figure 4E), and disappear (Figure 4F)—that represent six kinds of change patterns of TAD boundaries in colorectal cancer cells compared to normal colon cells. Previous studies have indicated that gene transcription is correlated with insulation at TAD boundaries [6,11]. Expand, fusion, and appearing TADs in colorectal cancer cells are associated with more interactions between enhancers and genes, whereas split, narrow, and disappearing TADs are associated with less interactions between some enhancers and genes in colorectal cancer cells. The sixty-five percent of changed TADs in colorectal cancer cells in comparison to normal colon cells suggests global alterations of gene regulations in colorectal cancer cells. We noticed that more than 50% (51–86%, except for the disappear group, which is 24%) of these changed TADs contain colorectal-cancer-specific enhancers (Figure 4A–F), which suggests important roles for colorectal-cancer-specific enhancers in the alterations of the gene regulation network. 

### 2.4. Transcriptome Change in Colorectal Cancer Cells Is Associated with Colorectal-Specific Enhancers

**Figure 5:**
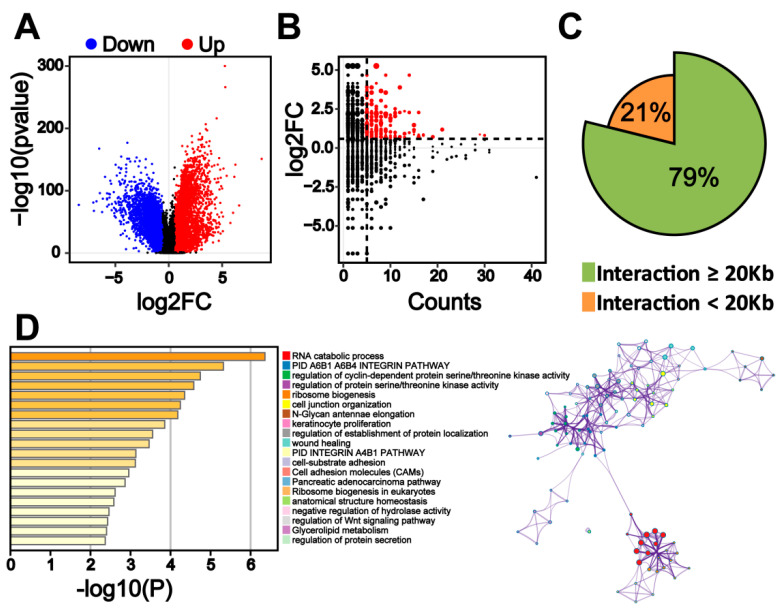
Transcriptome change in colorectal cancer cells is associated with colorectal-specific enhancers. (**A**) Volcano plot depicting gene expression changes in normal colon tissue (*n* = 349) and colorectal cancer tissue (*n* = 275). (**B**) Comparison of fold changes in gene expression based on RNA-seq and HiChIP signals. The plot shows 735 target genes. Red dots indicate target genes that were found to significantly change (fold change > 1.5; q < 0.01) in colorectal cancer tissue compared to those of normal colon tissue and have significant HiChIP signals (counts ≥ 5). (**C**) Percentage of long-range (>20 kb) interaction loops between colorectal-cancer-specific enhancers and target genes. (**D**) Functions of genes as analyzed by Metascape.

To determine whether the transcription change of genes in colorectal cancer cells is associated with colorectal-specific enhancers, we analyzed the transcriptome data of colon tissues (*n* = 349) and colorectal cancer tissues (*n* = 275) from TCGA. We found 4714 genes to be significantly upregulated (fold change > 1.5; q < 0.01) and 4924 genes to be significantly downregulated (fold change > 1.5; q < 0.01) in colorectal cancer tissue (Figure 5A). The combination analysis of RNA-seq and HiChIP data identified 152 genes that are significantly upregulated (fold change > 1.5; q < 0.01) in colorectal cancer tissue and have comparatively high interaction signals (interaction counts ≥ 5) with colorectal-cancer-specific enhancers (Figure 5B). These genes are potential target genes that are directly regulated by colorectal-cancer-specific enhancers. About 79% of the interactions between colorectal-cancer-specific enhancers and target genes are long-range (≥20 kb) interactions (Figure 5C). A pathway and process enrichment analysis by Metascape [19] showed that potential target genes of colorectal-cancer-specific enhancers are enriched in the RNA catabolic process, the α6β1α6β4 integrin pathway, and the regulation of cyclin-dependent protein serine/threonine kinase activity (Figure 5D). These pathways or biological processes are associated with colorectal cancer progression [20,21,22,23,24]. 

**Figure 6:**
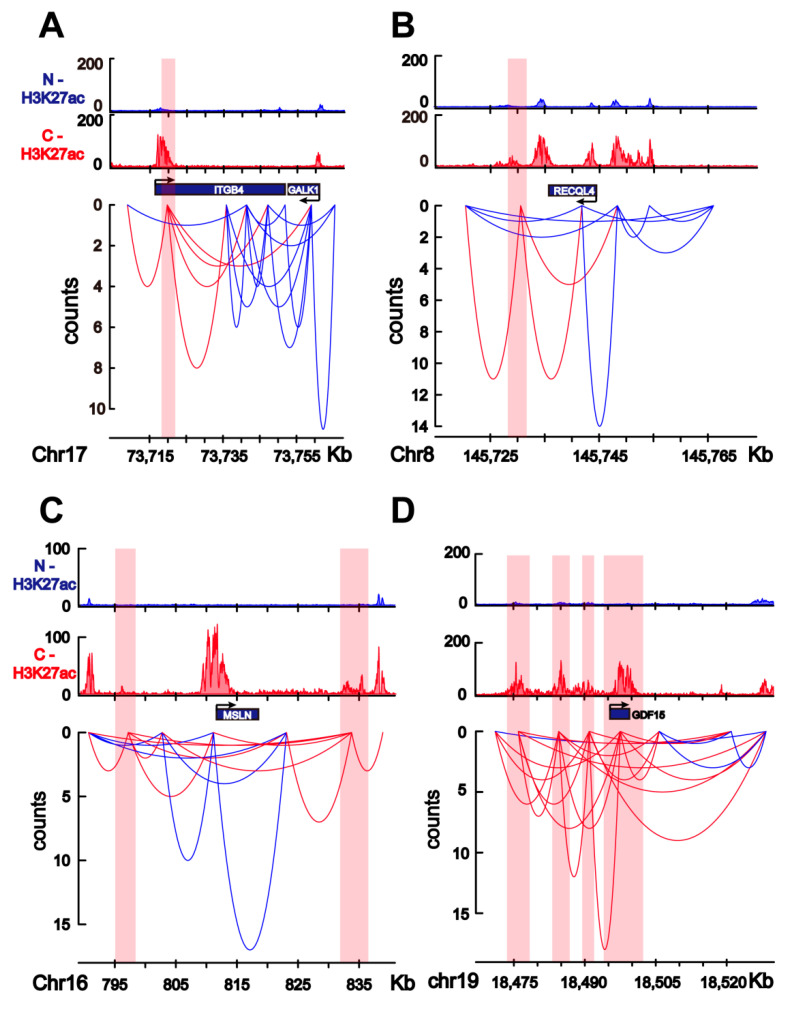
Target genes of the colorectal-cancer-specific enhancers. (**A**) H3K27ac enrichment for normal colon cells (N; blue) and colorectal cancer cells (C; red), as well as HiChIP interaction loops (red curves) between the *ITGB4* target gene and colorectal-cancer-specific enhancers. Colorectal-cancer-specific enhancers are shaded in red. Blue curves indicate other interaction loops in this area. (**B**) H3K27ac enrichment for normal colon cells (N; blue) and colorectal cancer cells (C; red), as well as HiChIP interaction loops (red curves) between the *RECQL4* target gene and colorectal-cancer-specific enhancers. The colorectal-cancer-specific enhancers are shaded in red. Blue curves indicate other interaction loops in this area. (**C**) H3K27ac enrichment for normal colon cells (N; blue) and colorectal cancer cells (C; red), as well as HiChIP interaction loops (red curves) between the *MSLN* target gene and colorectal-cancer-specific enhancers. Colorectal-cancer-specific enhancers are shaded in red. Blue curves indicate other interaction loops in this area. (**D**) H3K27ac enrichment for normal colon cells (N; blue) and colorectal cancer cells (C; red), as well as HiChIP interaction loops (red curves) between he *GDF15* target gene and colorectal-cancer-specific enhancers. Colorectal-cancer-specific enhancers are shaded in red. Blue curves indicate other interaction loops in this area.

*ITGB4* encodes integrin subunit β4 and is involved in the α6β1 α6β4 integrin pathway. HiChIP data showed that *ITGB4* is regulated by a colorectal-cancer-specific enhancer (Figure 6A), and RNA-seq data showed that *ITGB4* is upregulated (fold change = 2.31) in colorectal cancer tissue, suggesting the regulation of *ITGB4* by colorectal-cancer-specific enhancers. Mutations and aberrant expression of cancer-driver genes affect key cellular functions and induce cancer occurrence. *RECQL4* is a cancer-driver gene [25] and potential target gene that is regulated by colorectal-cancer-specific enhancers (Figure 6B). In addition, *RECQL4* is one of the most often mutated genes in colorectal cancer [26], and it is upregulated (fold change = 4.10) in colorectal cancer tissues. The colorectal-cancer-specific enhancer is about 15 kb away from the *RECQL4* promoter, and HiChIP data showed interactions between colorectal-cancer-specific enhancers and *RECQL4* (Figure 6B). In addition, *MSLN* and *GDF15* were found to be significantly upregulated (fold change = 12.24 for *MSLN* and fold change = 14.76 for *GDF15*) in colorectal cancer tissues, as well as to interact with colorectal-cancer-specific enhancers in colorectal cancer cells (Figure 6C, D). Mesothelin (MSLN) is reportedly associated with the prognosis of colorectal cancer [27], and growth differentiation factor 15 (GDF15) is reportedly associated with the recurrence and 5-fluorouracil resistance of colorectal cancer [28,29,30]. ChIP and HiChIP data showed that several colorectal-specific enhancers interact with *MSLN* and *GDF15*, and some of these enhancers interact with *MSLN* or *GDF15* through long-range chromatin interactions (>20 kb) (Figure 6C,D).

## 3. Discussion

This study shows that colorectal-specific enhancers interact with several potential target genes that are upregulated in colorectal cancer cells compared with normal colon cells, which is consistent with a previous study that show changes in the epigenome at enhancers are associated with transcriptional program to promote colon carcinogenesis [17]. Our study further unveils the regulation network of the colorectal-cancer-specific enhancers in the view of higher-order chromatin structures. HiChIP data indicated that higher-order chromatin structures induce interactions between colorectal-cancer-specific enhancers and target genes. Most altered TADs in colorectal cancer genome contain colorectal-specific enhancers. However, it is unclear whether changes in chromatin topological structures or the activation of colorectal-specific enhancers comprise the primary event in the initiation of colorectal cancer. A previous study showed that chromosomal topological changes repress stemness and invasion programs; these changes may restrain the malignant progression of colorectal cancer, and tumor-associated epigenomic changes are primarily oncogenic [11]. HiChIP data showed that some target genes of colorectal-cancer-specific enhancers are cancer-driver genes, including *RECQL4* (shown in Figure 6B), and an analysis of Hi-C data indicated that most boundary-changed TADs contain colorectal-specific enhancers (Figure 4). It is possible that the activation of colorectal-specific enhancers occurs before the alteration of higher-order chromatin structures during the occurrence and progression of colorectal cancer. Further studies are needed to verify this hypothesis.

With the development of chromatin conformation capture techniques [31,32,33,34], the analysis of higher-order chromatin structures in cancer cells has revealed complicated long-range gene networks of enhancers and indicated important functions of these enhancers or gene networks in cancer progression [8,9,10,13]. A recent study reported that mutations in enhancer loci lead to activity changes in the enhancer, affect the expression of the long-range target genes of this enhancer, and further improve the progression of prostate cancer [8]. A previous study showed that ependymoma enhancer profiles are distinct from other tissues and most super enhancers are tumor-specific and enriched with cancer-associated genes [13], suggesting important functions of these tumor-specific enhancers in the initiation of tumors. We profiled the colorectal-cancer-specific enhancers and presented the regulation networks of these enhancers based on HiChIP data, and RNA-seq data further unveiled the initiation and progression mechanisms of colorectal cancer. 

The combination analysis of HiChIP data and RNA-seq data indicated several potential target genes of colorectal-specific enhancers. *ITGB4* is one of these potential target genes. ITGB4 is one of the integrin (ITG) molecules that is associated with cell migration, proliferation, and cancer development [35,36,37]. Aberrant *ITGB4* expression has been reported in several cancers, including colorectal cancers [21,38]. RNA-seq analysis has also shown the significant upregulation of *ITGB4* in colorectal cancer tissue. A recent study indicated that ITGB4 might be a prognosis marker for the individual therapy of colon cancer [21]. Data in our study suggested that the upregulation of *ITGB4* in colorectal cancer cells may due to the regulation of colorectal-cancer-specific enhancers. We also noticed that *GALK1*, which is beside *ITGB4*, may be one of the potential target genes of the colorectal-cancer-specific enhancer that is shown in Figure 6A. HiChIP data indicated interactions between the colorectal-cancer-specific enhancer and *GALK1*, and RNA-seq analysis showed upregulation (fold change = 1.97; q = 7.76 × 10^−49^) of *GALK1* in colorectal cancer tissue compared to that in normal colon tissue. However, the interaction signal was not shown to be robust in the HiChIP data (count = 3). This may have been due to the long-range interactions between the colorectal-cancer-specific enhancer and *GALK1* (~45 kb). Our study suggests that *GALK1* may be regulated by colorectal-cancer-specific enhancers and be associated with the progression of colorectal cancer. However, few studies have reported the function of *GALK1* in colorectal cancer until now. 

*RECQL4* is an example of a cancer-driver gene that is regulated by colorectal-cancer-specific enhancers. RECQL4 was reported to be a DNA helicase and to function in DNA replication, repair, and recombination [39], and it plays an important role in maintaining genomic stability [40]. Several studies have indicated that RECQL4 is associated with cancer progression and may be a diagnostic marker for cancer [40,41,42,43,44], e.g., the overexpression of *RECQL4* is associated with a poor prognosis of gastric cancer [42] and predicts poor prognosis in hepatocellular carcinoma [43]. An investigation of the mutation spectrum of cancer-associated genes in patients with early onset colorectal cancer showed that *RECQL4* is one of the most often mutated genes [26]. Additionally, the present analysis of RNA-seq data from 275 colon cancer tissues and 349 normal colon tissues showed that *RECQL4* is upregulated in colorectal cancer cells. Based on the literature and the data analysis in the present study, *RECQL4* may be an important target gene of colorectal-cancer-specific enhancers and involved in the progression of colorectal cancer. HiChIP data also indicated that *MLSN* and *GDF15* interact with colorectal-specific enhancers through long-range chromatin interactions. MSLN is a cell surface protein that is reportedly highly expressed in several types of malignant tumors, including colorectal cancer [27]. Highly expressed MSLN induces an increase in cell proliferation in colorectal cancer, as well as worse prognosis [27]. In addition, MSLN was reported as one of the top cell therapy targets for tumors [45]. The upregulation of *GDF15* was reported correlate with an increased risk of recurrence and a decreased overall survival of colorectal cancer [28]. Our study suggests that the target genes of colorectal-cancer-specific enhancers may be potential targets for treating colorectal cancer. Methods such as the use of zinc-finger proteins and dCas9 to modify chromatin structures or manipulate specific enhancer–promoter interactions have been reported [46,47,48,49], indicating the potential to treat cancer by focusing on 3D chromatin structures. Our study suggests that the chromatin interactions of colorectal-cancer-specific enhancers and their target genes can be potential new targets to treat colorectal cancer. The analyses in this study were mainly based on database mining and in vitro HiChIP data from one colorectal cancer cell line, so more in vitro/vivo experiments on larger scales of human tissue samples and mouse models are needed to further validate the regulations of specific target genes by the colorectal-cancer-specific enhancers, as well as further unveil the whole regulation network of colorectal-cancer-specific enhancers. Though further studies are needed to pinpoint whether there are key driver genes in the colorectal-cancer-specific enhancer regulation network or the progression of colorectal cancer is a combined result of these target genes, our study suggests important functions of colorectal-cancer-specific enhancers in colorectal cancer progression and that these enhancers may be potential therapeutic targets of colorectal cancer.

## 4. Materials and Methods

### 4.1. Cell Culture

The colorectal cancer cell line HCT116 was obtained from the ATCC. HCT116 cells were incubated at 37 °C with 5% CO_2_ and cultured in Dulbecco’s Modified Eagle Medium (GIBCO) supplemented with 10% fetal bovine serum (FBS) (BI) and 1% penicillin-streptomycin (GIBCO). 

### 4.2. HiChIP

HiChIP was performed as previously described with modifications [33]. Briefly, cells were crosslinked with 1% formaldehyde and lysed. Then, the chromatin was digested using *Mbo*I (NEB), and the restricted ends were ligated by T4 ligase (NEB). Pelleted nuclei were dissolved in a nuclear lysis buffer (50 mM Tris-HCl, pH 7.5, 10 mM EDTA, 1% SDS, and protease inhibitors), and they were sonicated and diluted in a ChIP Dilution Buffer (0.01% SDS, 1.1% Triton X−100, 1.2 mM EDTA, 16.7 mM Tris-HCl, pH 7.5, and 167 mM NaCl). Then, immunoprecipitation was performed overnight at 4 °C by incubating H3K27ac antibodies (Abcam, Cambridge, UK) precoated on protein A-coated magnetic beads (Thermo Fisher Scientific, Waltham, MA, USA). Immunocomplexes were washed three times each with a low-salt buffer (0.1% SDS, 1% Triton X−100, 2 mM EDTA, 20 mM Tris-HCl, pH 7.5, and 150 mM NaCl), a high-salt buffer (0.1% SDS, 1% Triton X−100, 2 mM EDTA, 20 mM Tris-HCl, pH 7.5, and 500 mM NaCl), and an LiCl buffer (10 mM Tris-HCl, pH 7.5, 250 mM LiCl, 1% NP−40, 1% Na-Doc, and 1 mM EDTA). Beads were resuspended in a DNA elution buffer (50 mM NaHCO_3_ and 1% SDS). After elution, ChIP samples were incubated with 10 mg/mL of proteinase K 4 h at 55 °C. Then, DNA was purified using AMPure XP Beads (Beckman, Brea, CA, USA). Streptavidin C1 beads were used to capture biotinylated DNA. QIAseq FX DNA Library Kits were used to generate the sequencing library, and HiChIP libraries were size-selected to 300–700 bp using AMPure XP beads (Beckman) and subjected to 2 × 50-bp paired-end sequencing on HiSeq XTen (Illumina, San Diego, CA, USA).

### 4.3. ChIP-Seq Data Processing and Super Enhancer Analysis

The mapping of ChIP-seq data (Table 1) to hg19 was performed using bowtie2 [50]. H3K27ac peak calling was performed using MACS1.4 with default parameters [51]. Peak calling was separately performed for each sample. Peaks that could not be identified in each colorectal cancer sample and peaks that appeared within the region surrounding ±2.5 kb of transcriptional start sites were excluded from any further analysis. Afterwards, the H3K27ac peaks of the 7 individual samples (7 published colorectal cancer H3K27ac CHIP-seq datasets from ENCODE [16] and a published study [17]) were merged into a single set of peaks. Super enhancers were identified using the rank ordering of super enhancers (ROSE) algorithm [14]. For colorectal-cancer-specific enhancers, we removed all peak regions that contained any overlap with a peak detected in each normal colon region.

### 4.4. Unsupervised Hierarchical Clustering Analysis

A matrix of the normalized H3K27ac density was generated using HOMER [52]. Variant enhancer loci (VELs) were defined as enhancers that exhibited the greatest median absolute deviation (MAD) across all samples used for clustering. After the unsupervised hierarchical clustering between colorectal cancer and Roadmap Epigenomics samples data, 11,463 VELs were retained. These enhancers were used for unsupervised hierarchical clustering using a Pearson correlation as a distance metric.

### 4.5. t-SNE Analysis

For the clustering of H3K27ac ChIP-seq data from the colorectal cancer and normal colon cohorts together, we generated a normalized H3K27ac density matrix for both cohorts. The distance between samples was calculated by using “1- Spearman correlation coefficient” as the distance measure. The resulting distance matrix was used to perform the *t*-SNE analysis (Rtsne package).

### 4.6. HiChIP Data Processing

Valid fragment pairs and interaction matrixes were generated using Hi-C-Pro [53]. Then, interaction loop calling was performed using the hichipper [54], and the resulting interaction loops in Figure 3B–E are shown as a Circos plot [55]. For colorectal-cancer-specific enhancer loops detection, we selected loops that contained anchors that overlapped with cancer-specific enhancers.

### 4.7. Hi-C Data Analysis 

Two HiC data sets in GSE133928, BRD3179 and BRD3179N [11], representing colorectal cancer tissue and normal colon tissue, respectively, were analyzed to detect TADs in colorectal cancer cells and normal colon cells. Expanded TADs or narrowed TADs refers to the boundaries of TADs in colorectal cancer cells that are expanded or narrowed, respectively, in comparison to normal colon cells. Fusion TADs refers to two or more TADs in normal colon cells fused to one TAD in colorectal cancer cells. In comparison to normal colon cells, new insulation boundaries were formed in one TAD in colorectal cancer cells; this is referred to as a split TAD. Appeared TADs refers to TADs in colorectal cancer cells that do not exist in normal colon cells. Disappeared TADs refers to TADs in normal colon cells that disappear in colorectal cancer cells. 

## 5. Conclusions

In summary, our study shows that colorectal-cancer-specific enhancers regulate several target genes including cancer-driver genes and may play important roles in the occurrence and progression of colorectal cancer.

## Figures and Tables

**Table 1:** ChIP-seq data sets.

Sample Class	NO.	Sample	Data Source
Normal Colon	1	E075-H3K27ac.broadPeak	Roadmap
	2	E101-H3K27ac.broadPeak	Roadmap
	3	E102-H3K27ac.broadPeak	Roadmap
	4	E106-H3K27ac.broadPeak	Roadmap
	5	ENCFF116PPS	ENCODE
	6	ENCFF154YYF	ENCODE
	7	ENCFF193RGE	ENCODE
	8	ENCFF345LME	ENCODE
	9	ENCFF389IUW	ENCODE
	10	ENCFF779JEH	ENCODE
Colorectal Cancer	1	V400_H3K27ac	GSE36204
	2	V429_H3K27ac	GSE36204
	3	V503_H3K27ac	GSE36204
	4	V9M_H3K27ac	GSE36204
	5	ENCFF436VPF	ENCODE
	6	ENCFF570UFJ	ENCODE
	7	ENCFF720SPY	ENCODE

## Data Availability

HiChIP data were deposited in the NCBI Gene Expression Omnibus (GEO, https://www.ncbi.nlm.nih.gov/geo/, accessed on 5 May 2021) under the accession number GSE173699. The following published datasets were used in our analysis: the GSE36204 dataset was used for H3K27ac ChIP-seq analyses [17]. Other ChIP-seq data were obtained from Roadmap Epigenomics [15] (http://www.roadmapepigenomics.org/, accessed on 24 December 2020) and ENCODE (https://www.encodeproject.org/, accessed on 24 December 2020) [16]. The GSE133928 dataset was used for Hi-C analysis (Hi-C data in colorectal cancer cells and normal colon cells) [11], and RNA-seq data were obtained from the TCGA and GEPIA database (http://gepia.cancer-pku.cn/, accessed on 10 January 2021) [56].
